# Characteristics of pediatric SARS-CoV-2 healthcare-associated infection outbreaks in Germany, 2020–2023: a retrospective observational study

**DOI:** 10.1007/s00431-025-06680-x

**Published:** 2025-12-18

**Authors:** L. E. Schneider, M. Brandl, A. Ullrich, B. Piening, T. Eckmanns, M. Diercke, B. Suwono, S. Haller

**Affiliations:** 1 Department of Infectious Disease Epidemiology, Robert Koch Institute, Berlin, Germany; 2 Postgraduate Training for Applied Epidemiology (PAE), Department of Infectious Disease Epidemiology, Robert Koch Institute, Berlin, Germany; 3 ECDC Fellowship Programme, Field Epidemiology Path (EPIET), European Centre for Disease Prevention and Control (ECDC), Stockholm, Sweden; 4 Charité – Universitätsmedizin Berlin, Corporate Member of Freie Universität Berlin and Humboldt-Universität Zu Berlin, Berlin, Germany

**Keywords:** COVID-19, Pediatrics, Health personnel, Disease outbreaks, Hospitals, SARS-CoV-2, Healthcare-associated infections

## Abstract

Limiting healthcare-associated infections (HAI) SARS-CoV-2 transmission has been a global challenge. We assessed characteristics of pediatric SARS-CoV-2 HAI outbreaks to better understand HAI transmission in pediatric care. We analyzed PCR-confirmed SARS-CoV-2 outbreak cases notified through statutory surveillance, at case level and at outbreak level, in Germany between 24 February 2020 and 31 December 2023 to describe SARS-CoV-2 HAI outbreaks in pediatric care and compare them with non-pediatric outbreaks. Pediatric outbreaks included any outbreak in pediatric care or with > 50% of cases among patients aged < 18 years. We calculated age-disaggregated median monthly incidences of SARS-CoV-2 HAI outbreak cases per 100,000 hospitalizations using reimbursement data. Among 36,371 SARS-CoV-2 HAI hospital outbreak cases from 3191 outbreaks, we identified 25 pediatric outbreaks with 241 cases. Of 218 cases with case status information, 181 (83%) were healthcare workers (HCW) and 37 (17%) were patients < 18 years old. Among all 241 cases, five (2%) were severe including one death case whereas 2580 (7%) of non-pediatric HAI cases were severe and 2736 (8%) deaths. Age-disaggregated median monthly outbreak case incidence < 18 years (0.3/100,000 hospitalizations; range 0.1–7.6) was markedly lower than among the non-pediatric age group of 18–59-year olds (14.8/100,000 hospitalizations; range 0.1–215.7).

*Conclusion*: Our results suggest limited SARS-CoV-2 HAI transmission in pediatric care. The high proportion of cases among HCW in pediatric outbreaks underlines the need to protect HCW from infections to further limit HAI transmission. Our findings highlight the value of outbreak surveillance for monitoring HAI transmission patterns in vulnerable sub-groups such as pediatric patients.

**Table:** 

**What is Known:**
• *Children and adolescents experience less severe SARS-CoV-2 illness*. • *Pediatric care poses unique infection prevention and control challenges*.
**What is New:**
• *Pediatric outbreaks were very rare with few severe cases suggesting that pediatric care was less affected by SARS-CoV-2 outbreaks*. • *Most cases in pediatric outbreaks occurred among healthcare workers pointing to the need to protect HCW from infections and a limited role of pediatric patients and caregivers*.

**Supplementary Information:**

The online version contains supplementary material available at 10.1007/s00431-025-06680-x.

## Introduction

Children and adolescents, generally understood as minors under 18 years old, were regarded as a key population of interest early in the severe acute respiratory syndrome coronavirus 2 (SARS-CoV-2) pandemic driven by concerns that children may be severely affected given their increased susceptibility to other respiratory illnesses [1]. In light of higher rates of asymptomatic infection among children, their contribution to transmission was heavily debated [2–4]. Today evidence indicates that compared to adults, children are less susceptible to SARS-CoV-2 infection and less likely to transmit the virus [5].

While measures such as universal masking, cohorting, visitor restrictions, and testing during the pandemic appear to have contributed to reductions in other respiratory viruses like RSV and influenza within pediatric hospitals [6], pediatric care remains a challenging setting to implement infection prevention and control (IPC) during respiratory infectious disease epidemics. Implementation of IPC measures such as isolation is complicated by considerations to ensure the well-being of pediatric patients through contact and support of caregivers who may unknowingly contribute to healthcare-associated infection (HAI) transmission [7, 8].

Results of seroconversion studies among healthcare workers (HCW) and genomic surveillance highlight the increased risk for SARS-CoV-2 infection among HCW, which was likely underestimated, and underline the importance of better understanding HAI transmission to best protect patients [9, 10]. At the same time, incidence rates among < 18 year olds were comparable with older age groups with incidence rates of more than 2000 infections per 100,000 population in Germany in early 2022, for example [11].

In Germany, pediatric patients are cared for in pediatric clinics or wards and throughout the pandemic the same guidance as for adults applied [12]. While single pediatric outbreaks have been reported [13–15] and insights into HAI transmission in pediatric care have been assessed in individual hospitals [16, 17], SARS-CoV-2 HAI outbreaks in pediatric care have to our knowledge not been comprehensively analyzed on a national level.

Since 2011 information on HAI outbreaks is systematically collected in Germany to facilitate outbreak investigations at local level as well as to monitor trends and patterns in outbreaks [18]. Insights on pediatric HAI outbreaks during the SARS-CoV-2 pandemic can help to inform our understanding of transmission dynamics and the role staff and patients may play in future respiratory epidemics. We describe pediatric SARS-CoV-2 HAI hospital outbreaks and case characteristics from 2020 to 2023 and compare them with non-pediatric HAI hospital outbreaks to better understand SARS-CoV-2 HAI transmission in pediatric care.

## Methods

In this retrospective observational study we used data from the German statutory surveillance to describe case characteristics of pediatric and non-pediatric SARS-CoV-2 outbreaks that occurred between 24 February 2020 and 31 December 2023 (with data extracted on 24 January 2024). We included all notified polymerase chain reaction (PCR)-confirmed SARS-CoV-2 infections. In line with the German surveillance outbreak definition [19], we defined a *SARS-CoV-2 outbreak* as at least two PCR-confirmed SARS-CoV-2 cases with an epidemiological link. Notification data is collected at the case level (case-based information) and outbreak level (general outbreak information). *SARS-CoV-2 HAI outbreaks* were defined as any outbreak reported by local health authorities as “healthcare-associated” (notified in accordance with Sect. 6 (3) of the German Infection Protection Act) and in addition all outbreaks with the setting “hospital” or where > 50% of outbreak cases were specified with the setting “hospital” following Suwono et al. [20]. As *SARS-CoV-2 HAI outbreak cases* we included associated cases that belonged to a SARS-CoV-2 HAI outbreak.

To describe outbreaks in pediatric care, we defined *SARS-CoV-2 HAI pediatric outbreaks* as outbreaks with the setting “pediatric ward” or “pediatric clinic” (Definition A, see Supplement 1). Due to limited completeness of this variable, we added all outbreaks where > 50% of cases were patients < 18 years (Definition B, see Supplement 1). We chose patients < 18 years old as they routinely receive care in specialized pediatric wards in Germany. We then defined *SARS-CoV-2 HAI pediatric outbreak cases* as all cases belonging to a pediatric SARS-CoV-2 HAI outbreak.

To describe the trend of SARS-CoV-2 HAI outbreak cases throughout the pandemic, we classified five pandemic phases in accordance with an established German classification used throughout the pandemic and one post-pandemic phase [21]. The start of the outbreak was defined as the notification date of the first case belonging to the outbreak.

We used several parameters to characterize pediatric and non-pediatric HAI outbreaks using summary statistics: 1) outbreak characteristics (size) 2) demographic case characteristics (age and administrative sex), 3) case status (HCW, patient, undefined), and 4) case fatalities (severe cases and deaths). Cases were considered severe if at least one of the following symptoms was reported: pneumonia, dyspnoea, acute respiratory distress syndrome (ARDS) or need for respiratory support.

To address changes in the number of hospitalizations during the SARS-CoV-2 pandemic, we calculated age-disaggregated median monthly incidence of SARS-CoV-2 HAI outbreak cases per 100,000 hospitalizations by age group between 2019 and 2023. We used the number of SARS-CoV-2 outbreak cases in each specified age group (< 18 years old, 18 – 59 years old and > 60 years old) and hospitalizations in the corresponding age group per month extracted from the national hospital reimbursement data. Hospitalization data was collected according to § 21 of the Hospital Remuneration Act (Krankenhausentgeltgesetz) and available through the German Institute for the Hospital Remuneration System (Institut für das Entgeltsystem im Krankenhaus, InEK) [22].

All descriptive analyses and visualizations were conducted with R (Version 4.3.0) [23] including packages rio, scales, flextable janitor, apyramid, tidyr, DiagrammeR, ggplot2 and dplyr [24–32].

## Results

**Fig. 1:**
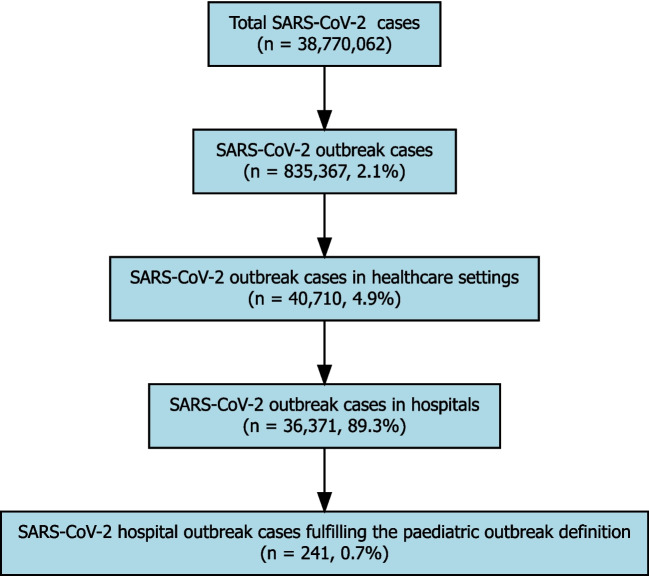
Flowchart of the outbreak definition stratification process with data from German statutory surveillance notified according to German Infection Protection Act between February 2020 and December 2023

Between 24 February 2020 and 31 December 2023, 38,770,062 SARS-CoV-2 cases were notified within Germany’s statutory surveillance system, see Fig. 1. Of these, 835,367 (2%) were linked to an outbreak, and among these, 40,710 (5%) were associated with a HAI outbreak. Thereof 36,371 cases (89%) were reported in hospitals.

**Fig. 2:**
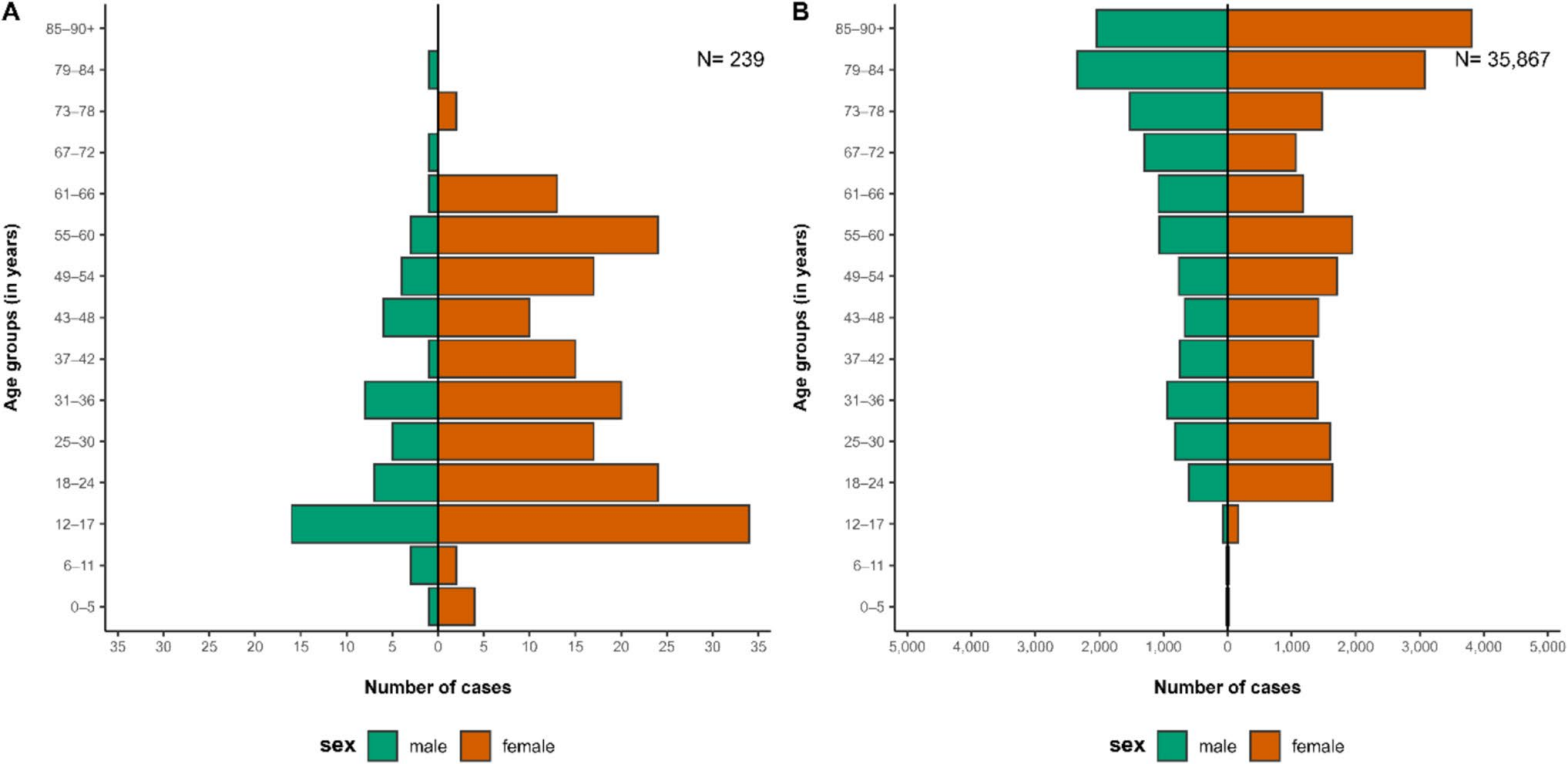
Age-sex distribution of SARS-CoV-2 cases in pediatric (**A**) and non-pediatric (**B**) healthcare-associated infection outbreaks in Germany, February 2020 to December 2023

**Fig. 3:**
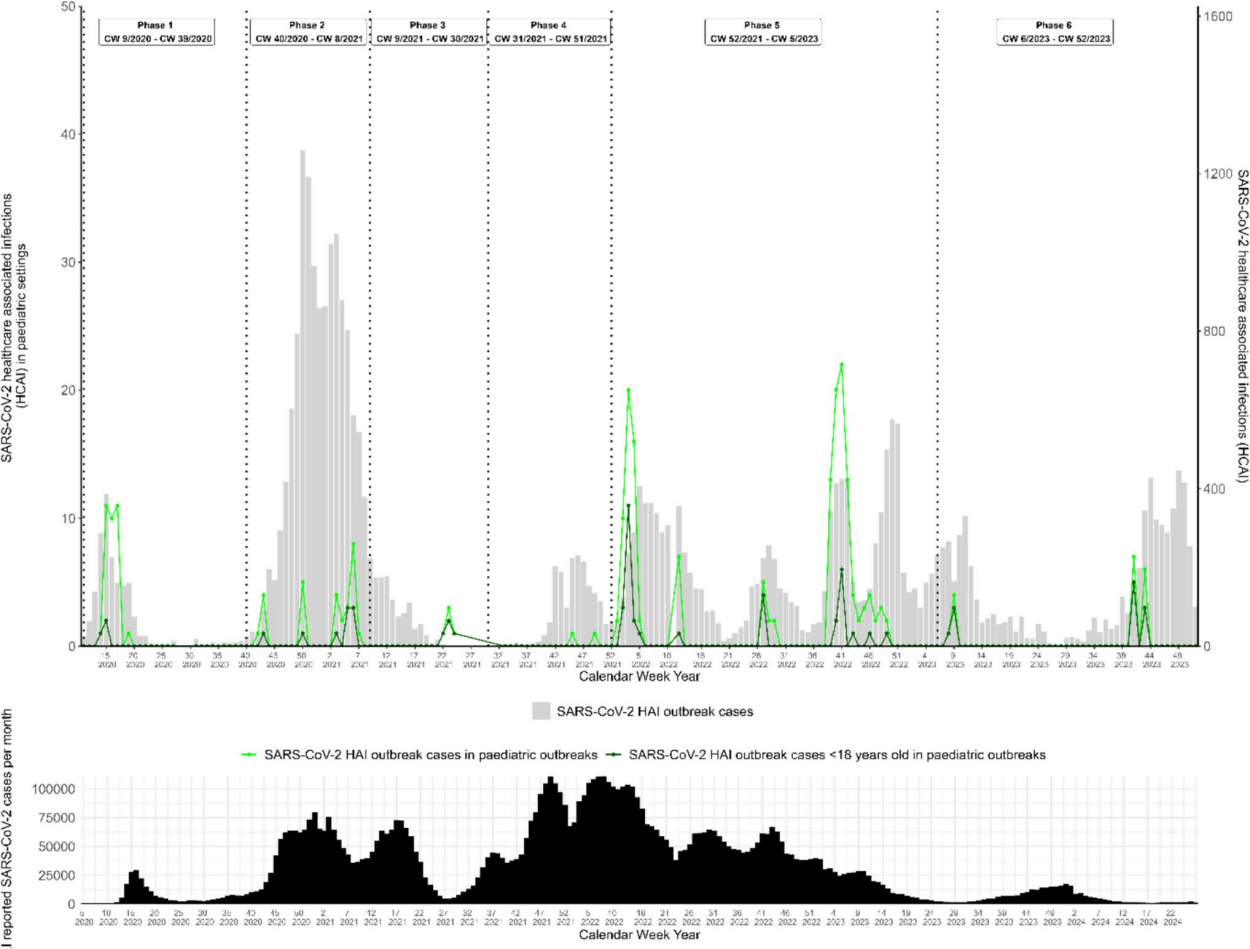
Pediatric SARS-CoV-2 healthcare associated infection outbreak cases in hospitals overall and < 18 years old in comparison to non-pediatric SARS-CoV-2 HAI with total reported SARS-CoV-2 infections below, Germany, February 2020 to December 2023

We identified 25 pediatric outbreaks according to our outbreak definition with a total of 241 associated cases and 3166 non-pediatric outbreaks with 36,130 associated cases, see Table 1. Among pediatric outbreak cases, 182 (76%) were females. Adults in these outbreaks were predominantly females (138, 79%), whereas among individuals < 18 years of age, 43 (67%) were females, see Fig. 2. Of pediatric outbreak cases < 18 years old, only five cases were < 6 years old and five cases between six and < 12 years old. For three cases no information on age was available. Among all cases belonging to pediatric outbreaks, 41 (17%) were patients, 181 (75%) were HCW and for 19 (8%) the status was undefined, whereas among the 61 pediatric outbreak cases < 18 years old, 37 (61%) were patients, 15 (25%) HCW and for nine (15%) the status was undefined, see Table 1. Five cases, all occurring before the end of 2021 (phase 5), were severe and one death was reported during the first half of 2020 (phase 1). No death occurred among < 18 year olds. Examining the number of outbreaks over time, only 8 out of 25 pediatric SARS-CoV-2 HAI outbreaks were reported by the end of 2021 (before phase 5), see Fig. 3.

**Table 1:** Non-pediatric and pediatric hospital SARS-CoV-2 hospital-acquired infection outbreaks and outbreak case characteristics in Germany by pandemic phase between February 2020 to December 2023

	Phase 1 (CW* 09/2020–39/2020)	Phase 2 (CW 40/2020–8/2021)	Phase 3 (CW 9/2021–30/2021)	Phase 4 (CW 31/2021–51/2021)	Phase 5 (CW 51/2021–5/2023)	Phase 6 (CW 6/2023–52/2023)	Total
**All SARS-CoV-2 cases**	175,285	2,270,030	1,326,595	3,189,531	30,612,099	1,196,522	38,770,062
**Non-pediatri**c** hospital** **outbreaks**							
Number of non-pediatric outbreaks	115	605	99	198	1301	848	3166
Median outbreak size (Q1 – Q3)	8 (6–28)	9 (6–29)	7 (5–9)	7 (5–8)	7 (5–8)	6 (5–8)	7 (5–9)
Number of outbreak cases	2293	13,187	920	2008	11,360	6362	36,130
Administrative sex							
Number of females (%)	1554 (67.8)	8601 (65.2)	550 (59.8)	1180 (58.8)	6522 (57.4)	3499 (55.0)	21,906 (60.6)
Number of males (%)	736 (32.1)	4550 (34.5)	370 (40.2)	807 (40.2)	4755 (41.9)	2824 (44.4)	14,042 (38.9)
Number of cases with unknown sex (%)**	3 (0.1)	35 (0.3)	0 (0)	21 (1)	83 (0.7)	39 (0.6)	182 (0.5)
Age							
Number of cases with age unknown (%)	1 (0)	11 (0.1)	0 (0)	1 (0)	81 (0.7)	74 (1.2)	168 (0.5)
Median age (Q1- Q3)	50 (33–68)	56 (35–78)	63 (41–81)	58 (38–79)	65 (42–82)	79 (65–85)	63 (41–81)
Number of cases < 18 years old (%)	26 (1.1)	179 (1.4)	9 (1)	26 (1.3)	57 (0.5)	7 (0.1)	304 (0.8)
Case status							
Number of patients (%)	200 (8.7)	1906 (14.5)	154 (16.7)	390 (19.4)	2557 (22.5)	1420 (22.3)	6627 (18.3)
Number of healthcare workers (%)	1126 (49.1)	4306 (32.7)	233 (25.3)	677 (33.7)	2375 (20.9)	266 (4.2)	8983 (24.9)
Number of cases with undefined status	967 (42.2)	6975 (52.9)	533 (57.9)	941 (46.9)	6428 (56.6)	4676 (73.5)	20,520 (56.8)
Severity and fatalities							
Number of cases with severe symptoms (%)	245 (10.7)	1210 (9.2)	93 (10.1)	163 (8.1)	458 (4.0)	411 (6.5)	2580 (7.1)
Number of deaths (%)	195 (8.5)	1569 (11.9)	148 (16.1)	212 (10.6)	386 (3.4)	226 (3.6)	2736 (7.6)
**Pediatric** **hospital** **outbreaks**							
Number of pediatric outbreaks	1	5	1	1	14	3	25
Median outbreak size (Q1 – Q3)	34 (-)	5 (5–5)	6 (-)	5 (-)	7 (6–8)	6 (6–7)	6 (5–8)
Number of outbreak cases	34	28	6	5	150	18	241
Sex							
Number of females (%)	22 (64.7)	24 (85.7)	6 (100)	1 (20)	118 (78.7)	11 (61.1)	182 (75.5)
Number of males (%)	12 (35.3)	4 (14.3)	0 (0)	4 (80)	32 (21.3)	6 (33.3)	58 (24.1)
Number of cases with unknown sex (%)	0 (0)	0 (0)	0 (0)	0 (0)	0 (0)	1 (5.6)	1 (0.4)
Age							
Number of cases with age unknown (%)	0 (0)	0 (0)	0 (0)	0 (0)	1 (0.7)	0 (0)	1 (0.4)
Median age (IQR)	34 (28–49)	23 (17–42)	15 (14–32)	16 (13–18)	35 (22–52)	16 (13–19)	32 (17–50)
Number of cases under 18 years old (%)	3 (8.8%)	9 (32.1%)	4 (66.7%)	3 (60%)	30 (20%)	12 (66.7%)	61 (25.3%)
Case status							
Number of patients (%)	1 (2.9)	0 (0)	5 (83.3)	3 (60)	19 (12.7)	13 (72.2)	41 (17)
Number of patients < 18 years old (%)	0 (-)	0 (-)	4 (66.7)	3 (60.0)	18 (12.0)	12 (66.7)	37 (15.4)
Number of healthcare workers (%)	32 (94.1)	23 (82.1)	1 (16.7)	0 (0)	120 (80.0)	5 (27.8)	181 (75.1)
Number of healthcare workers < 18 years old (%)	3 (8.8)	5 (17.9)	0 (-)	0 (-)	7 (4.7)	0 (-)	15 (6.2)
Number of cases with unknown status (%)	1 (2.9)	5 (17.9)	0 (0)	2 (40)	11 (7.3)	0 (0)	19 (7.9)
Severity and fatalities							
Number of cases with severe symptoms (%)	2 (5.9)	1 (3.6)	1 (16.7)	1 (20)	0 (0)	0 (0)	5 (2.1)
Number of deaths (%)	1 (2.9)	0	0	0	0	0	1 (0.4)

Data was extracted from the German notification system on 26 January 2024

^*^Calendar weeks (CW) refer to standardized, numbered weeks of the year (e.g., CW01–CW52) with time intervals starting on Mondays

^**^Including one case classified as gender-diverse (German: divers)

Comparing pediatric SARS-CoV-2 HAI outbreaks to non-pediatric SARS-CoV-2 HAI outbreaks, SARS-CoV-2 HAI pediatric outbreaks included a higher proportion of females (76% vs. 61%) and HCW (75% vs. 25%), see Table 1. Completeness of case status information was overall lower in non-pediatric outbreaks with 57% of information on status as HCW or patient missing in comparison to 8% with missing information among pediatric outbreak cases. Among the 304 cases < 18 years old in non-pediatric outbreaks, 235 (77%) were 12–17 years old in comparison to 51 (84%) cases in pediatric outbreaks. Symptoms from SARS-CoV-2 illness in pediatric outbreak cases were less often severe with five (2%) and only one (0.4%) death, compared to 2580 (7%) being severe and 2736 deaths (8%) among non-pediatric outbreak cases. In comparison, 18,408 (51%) of non-pediatric outbreak cases but only 73 (30%) pediatric HAI outbreak cases occurred by the end of 2021 (before phase 5), see Fig. 3.

**Fig. 4:**
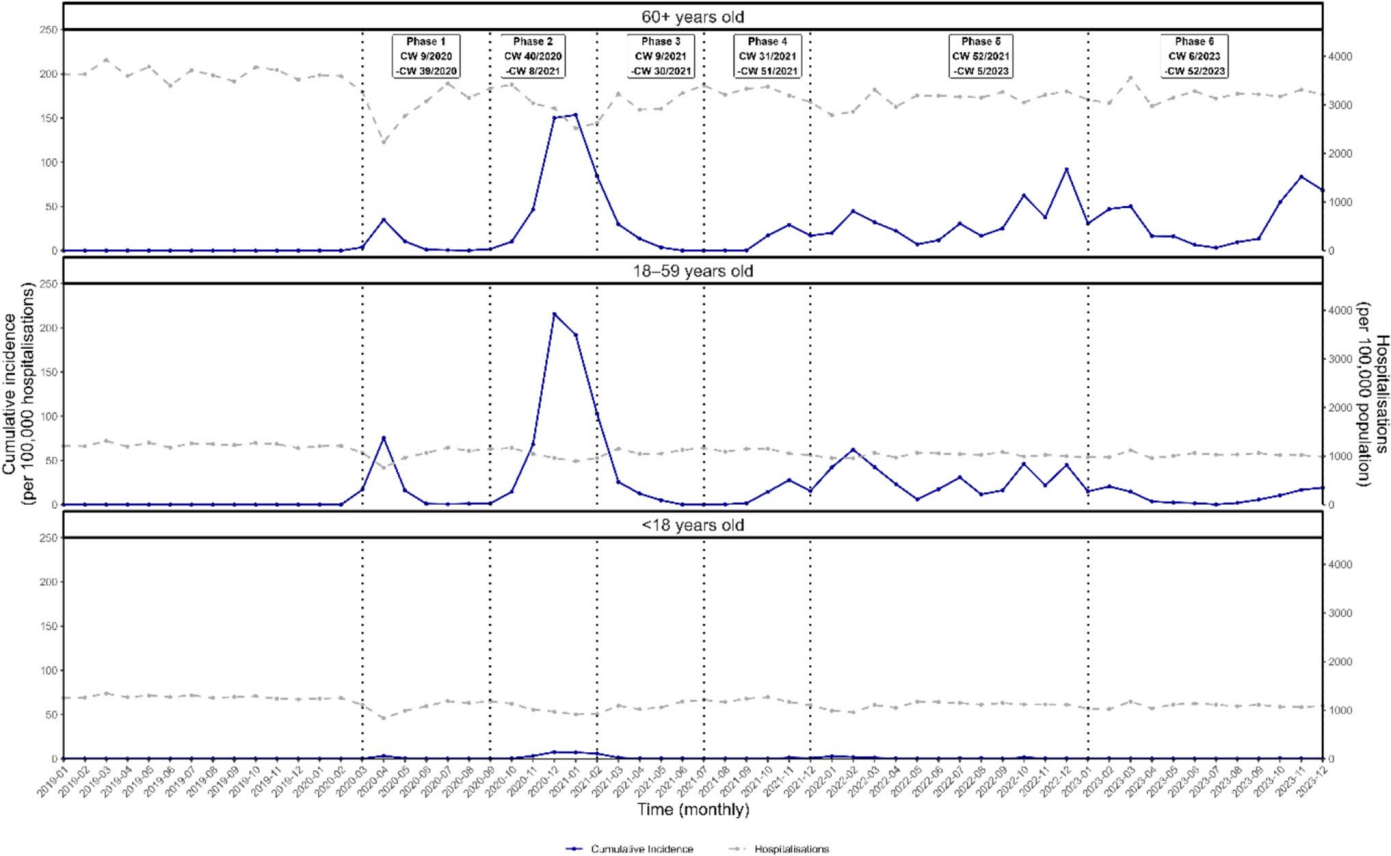
Age-disaggregated monthly incidence of SARS-CoV-2 HAI outbreak cases per 100,000 hospitalizations (all causes) and 100,000 hospitalizations (all causes), 2019–2023, Germany

The median monthly incidence was 0.3 SARS-CoV-2 HAI outbreak cases < 18 years old per 100,000 hospitalizations (range 0.1–7.6) in the same age group, see Fig. 4. In contrast, monthly incidence was significantly higher in older age groups, namely between 18 – 59 and > 60 years old, with a median incidence of 14.8 and 17 SARS-CoV-2 HAI outbreak cases per 100,000 hospitalizations, respectively (range, 0.1–215.7 and 0.1–153.5 respectively). Throughout the study period, the monthly incidence of SARS-CoV-2 HAI outbreak cases per 100,000 hospitalizations was lower in the age group of < 18 years old than in the other age groups.

## Discussion

Our results indicate that SARS-CoV-2 HAI outbreaks in pediatric care were rare, with few severe cases and only one death, and less frequent compared with non-pediatric outbreaks. The low incidence of SARS-CoV-2 outbreak cases per 100,000 hospitalizations in the age group of < 18 year olds indicates that the low occurrence of pediatric HAI outbreaks cannot be explained by changes in hospitalizations due to pandemic measures alone, but likely reflects a real difference in the frequency of outbreaks in pediatric and non-pediatric care. While these findings align with literature suggesting a limited role of children in transmission dynamics [33], the high incidence among children in the general population raises questions about the low number of pediatric HAI outbreaks. Among other reasons underdetection and misclassification need to be taken into account [11].

We found only few severe cases in pediatric HAI outbreaks and none among children and adolescents under < 18 years old. This observation is consistent with reports of rare severe outcomes of SARS-CoV-2 infections in this age group [34]. As children and adolescents with SARS-CoV-2 were rarely hospitalized with one systematic review reporting a hospitalization rate of 3.3% among children testing positive [34], the possibility of exposure to SARS-CoV-2 and consequently infection was likely lower in pediatric care due to fewer introductions of SARS-CoV-2.

With 304 cases, a considerable fraction of SARS-CoV-2 HAI outbreak cases < 18 years old occurred in outbreaks that we categorized as non-pediatric outbreaks which could point to a misclassification of outbreaks. Due to missing specification of settings to the detail of pediatric clinic or ward (following Definition A), we additionally analyzed different thresholds for inclusion of cases < 18 years old. Following Definition B (Supplement 1^1^) we include all outbreaks where > 50% of cases were < 18 years old, with a 50% threshold as a conservative criterion. Using a lower threshold (e.g., 40% or 30%, see Definition C and D in Supplement 1) would have substantially increased the number of outbreaks classified as pediatric (from 241 to 498 when lowering the threshold to 30% (Definition D), for example; Supplement 1), but at the cost of likely misclassifying general-ward outbreaks with incidental pediatric cases. Thus, our approach prioritizes specificity over sensitivity to ensure that outbreaks identified as pediatric reflect pediatric care contexts. Overall SARS-CoV-2 HAI cases < 18 years old are very rare and only represent 1% and thus a small fraction of cases and even with broader definitions pediatric outbreaks remain rare. Cases < 18 years old in non-pediatric outbreaks likely present individual outbreak cases in outbreaks across wards. As most of them were aged between 12 and 17 years, some may have been HCW in training as in Germany vocational nursing training is open from 16 years as also observed in pediatric outbreaks [35]. The overlap in age between HCW and patients in pediatric outbreaks is a unique characteristic challenging description and classification of pediatric outbreaks. Adolescents may also receive care for in non-pediatric care due to clinical considerations.

HCW have been shown to play a central role in SARS-CoV-2 HAI outbreaks [36]. The high number of female adults and HCW in pediatric outbreaks in comparison to non-pediatric outbreaks in our study suggests that HCW, particularly nurses of who according to data from 2019 89.4% are female in Germany [37], may have played a central role in SARS-CoV-2 HAI transmission in pediatric care. This may indicate that, where transmission occurred, it might have been driven by staff-to-staff spread thus exemplifying the importance of protection HCW from infection and compliance with IPC protocols.

Next to HCW caregivers, often close relatives, are critical in the provision of adequate pediatric care. As caregivers are not identifiable directly from the statutory surveillance data, their role in transmission could be underestimated. With all but 19 cases reported as neither patients or HCW, the number of cases that could represent caregivers is limited indicating a minor contribution at most. While the proportion of cases with undefined status is highest in phase 2, the number of cases with undefined status remains small, ranging from 1 to 11 and no temporal clustering can be observed.

Non-pediatric SARS-CoV-2 HAI outbreak cases showed a clear peak at the beginning of the pandemic during phase 2 in the first winter wave in 2020/2021[20, 38]. Among others, high rates of hospitalization, limited guidance, and susceptibility to a new variant of the virus may explain this observation. Unfortunately we do not have data allowing the description of SARS-CoV-2 variants on individual level or outbreak level. Ecological studies suggest that certain variants were associated with higher or lower morbidity and mortality [39]. Interestingly, pediatric outbreaks and associated cases did not follow this trend with most pediatric outbreaks reported from 2022 onwards. The exploration of underlying reasons goes beyond this study as various factors may account for this observation including, among others, relaxed measures, high vaccination rates in the adult population, high incidence rates during the omicron wave, higher capacity at local public health authority level and SARS-CoV-2 testing protocols for hospitals. While changes in public health and mitigation measures, such as mask mandates, quarantine requirements, and school closures, likely influenced SARS-CoV-2 transmission dynamics in the general population [40], federal implementation of non-pharmaceutical measures in Germany and their overlap across pediatric and non-pediatric care settings do not allow us to directly attribute differences in outbreak occurrence to specific measures. Recommendations for IPC in hospitals were relatively stable throughout the pandemic [12]. It remains unclear whether the relatively low occurrence of SARS-CoV-2 HAI pediatric outbreaks is specific to SARS-CoV-2 or whether similar observations may apply to other respiratory viruses. In general, infectious diseases present a higher burden in pediatric in-patient care. For instance, HAI detection rates of RSV were highest among infants and young children [41, 42]. During the pandemic, studies documented an overall decline of respiratory pathogens beyond SARS-CoV-2 which might indicate that effective infection prevention and control in pediatric care successfully reduced HAI outbreaks [6, 16]. This underlines the possibility to learn from effective IPC measures in pediatric care during the pandemic to prevent HAI outbreaks. Further epidemiological studies are needed to inform whether setting and specific pediatric care practices such as lower room density, staff-to-patient ratio, isolation and cohorting practices could benefit non-pediatric care during future epidemics.

The following limitations should be considered: firstly, for outbreaks in which the specific type of clinic, e.g., pediatric clinic was not reported, we defined an outbreak as pediatric if > 50% of outbreak cases were patients < 18 years old, which may have led to outbreak misclassification. We explored the extent to which outbreaks captured pediatric cases in a sensitivity analysis of the applied pediatric outbreak definition, see Supplement 1. Secondly, although there were no differentiated testing strategies between adults and children in hospital, it cannot be excluded that in practice testing strategies differed between pediatric and non-pediatric care leading to an underdetection of outbreaks in pediatric care. Thirdly, children might have had more asymptomatic infections of SARS-CoV-2 which could have further contributed to underdetection. Infection screening measures were implemented at the time of hospital admission, regularly for HCW and systematically when SARS-CoV-2 cases occurred on wards until early 2023 [12]. Fourthly, during periods with higher case load, increasing workload in local health authorities may have led to incompleteness of data entries, possibly leading to an underestimation of HAI outbreaks during these periods. Particularly additional variables such as reported symptoms and status as healthcare worker or patient were often missing information, see Table 1. This limitation is particularly pronounced when examining data completeness of case status (e.g. HCW, patient or undefined), where 57% of cases had an undefined status in non-pediatric SARS-CoV-2 HAI outbreaks, compared to only 8% in pediatric SARS-CoV-2 HAI outbreaks. This difference in completeness limits the reliability of direct comparisons of HCW and patient proportions between outbreak types and should therefore be interpreted with caution. While it is difficult to understand what influences data completeness, the rare occurrence of outbreaks in pediatric clinics and high concern for vulnerable pediatric patients may explain differences in reporting and investigation practices. The consistency of our findings across pediatric and non-pediatric outbreaks, as they relate to the rare occurrence of cases in < 18 years old in general, rare pediatric outbreaks and overall lower median monthly incidence of SARS-CoV-2 HAI outbreak cases per 100,000 hospitalizations in < 18 year olds, support the robustness of our results.

## Conclusion

Our study indicates that pediatric SARS-CoV-2 HAI outbreaks were rare and included very few severe cases. This suggests that pediatric care was less impacted than other settings by SARS-CoV-2 HAI outbreaks in Germany. Among pediatric SARS-CoV-2 HAI outbreaks, the proportion of cases among healthcare personnel was higher than in non-pediatric SARS-CoV-2 HAI outbreaks. In future epidemics, particular focus should be on protecting HCW from infection to further limit HAI transmission. Our study demonstrates the usefulness of outbreak surveillance to complement case-based statutory surveillance systems to offer insights into outbreak dynamics among vulnerable groups such as pediatric patients.

## Supplementary Information

Below is the link to the electronic supplementary material.

ESM 1 (DOCX 15.7 KB)

## Data Availability

The data from mandatory notification system has limited and restricted access. Further detailed information on outbreak data is protected under German law.

## Abbreviations

HAI: Healthcare-associated infection
HCW: Healthcare workers
IPC: Infection prevention and control
PCR: Polymerase chain reaction
SARS-CoV-2: Severe acute respiratory syndrome coronavirus 2

## Declarations

### Disclaimer

Lena E. Schneider is a fellow of the German Postgraduate Training in Applied Epidemiology (PAE) associated with the ECDC Fellowship Programme. The views and opinions expressed herein do not state or reflect those of ECDC. ECDC is not responsible for the data and information collation and analysis and cannot be held liable for conclusions or opinions drawn.

### Statement on AI

ChatGPT (version GPT-4o) was used to refine the R code for the statistical analysis, as well as for language editing without affecting the analysis or conclusions. The authors retain full responsibility of the content of the manuscript. Participants’ confidentiality was guaranteed by not sharing any sensitive information.
