# Supplementary material for: Characteristics of pediatric SARS-CoV-2 healthcare-associated infection outbreaks in Germany, 2020–2023: a retrospective observational study
Source: Eur J Pediatr. 2025 Dec 18;185(1):25. doi: 10.1007/s00431-025-06680-x (PMC12711983; doi:10.1007/s00431-025-06680-x)
Supplement: Supplementary file 1 — (DOCX 15.7 KB) [file 431_2025_6680_MOESM1_ESM.docx]

Title: Characteristics of pediatric SARS-CoV-2 healthcare-associated infection outbreaks in Germany, 2020 –2023: a retrospective observational study

Authors: L. Schneider, M. Brandl , A. Ullrich , B. Piening^4^, T. Eckmanns , M. Diercke , B. Suwono , S. Haller

Journal: European Journal of Pediatrics

Corresponding author: Lena Schneider, Department of Infectious Disease Epidemiology, Robert Koch Institute, Berlin, Germany, [schneiderl@rki.de](mailto:schneiderl@rki.de)

**Supplementary Information**

Supplementary Table 1: Number of notified SARS-CoV-2 HAI cases <18 years old included in different possible pediatric HAI SARS-CoV-2 outbreak definitions.

| **Pediatric outbreak definitions** | **Phase 1** | **Phase 2** | **Phase 3** | **Phase 4** | **Phase 5** | **Phase 6** | **Total** |
| --- | --- | --- | --- | --- | --- | --- | --- |
| Total SARS-CoV-2 HAI hospital outbreak cases | 2,327 | 13,215 | 926 | 2,013 | 11,510 | 6,380 | 36,371 |
| SARS-CoV-2 HAI hospital outbreak cases among patients <18 years old* | 29 | 188 | 13 | 29 | 87 | 19 | 365 |
| Pediatric outbreaks (combined definition: A + B) | **34** | **28** | **6** | **5** | **150** | **18** | **241** |
| Definition A) Notified as pediatric ward outbreak | 0 | 0 | 0 | 0 | 17 | 6 | 23 |
| Definition B) ≥50% of outbreak cases are <18 years old | 34 | 28 | 6 | 5 | 141 | 18 | 232 |
| Definition C) ≥40% of outbreak cases are <18 years old | 40 | 81 | 6 | 23 | 180 | 18 | 348 |
| Definition D) ≥30% of outbreak cases are <18 years old | 40 | 145 | 6 | 34 | 250 | 23 | 498 |
| Definition E) ≥2 cases in outbreak are <18 years old | 436 | 2,408 | 27 | 123 | 138 | 18 | 3,150 |
